# Supplementary material for: Efficient Environmentally Friendly Flexible CZTSSe/ZnO Solar Cells by Optimizing ZnO Buffer Layers
Source: Materials (Basel). 2023 Apr 4;16(7):2869. doi: 10.3390/ma16072869 (PMC10095816; doi:10.3390/ma16072869)
Supplement: Supplementary file 1 [file materials-16-02869-s001.zip › materials-2266048-supplementary.pdf]

Surporting information

Efficient Environmental Flexible CZTSSe/ZnO Solar Cells by Optimizing ZnO Buffer Layers

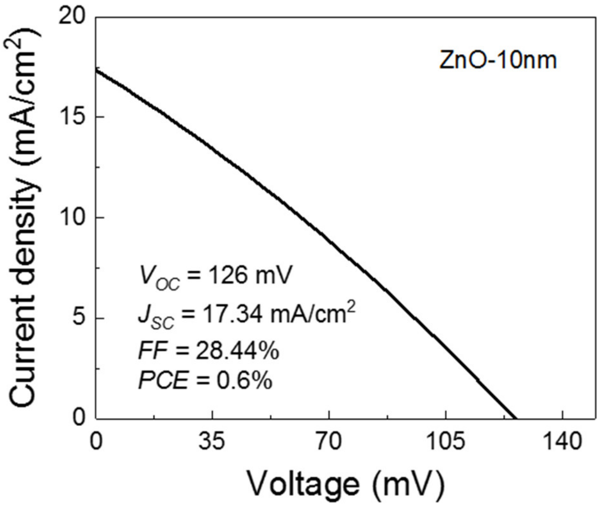

Figure S1. J-V curves of the flexible CZTSSe solar cells with 10 nm thick ZnO layers.

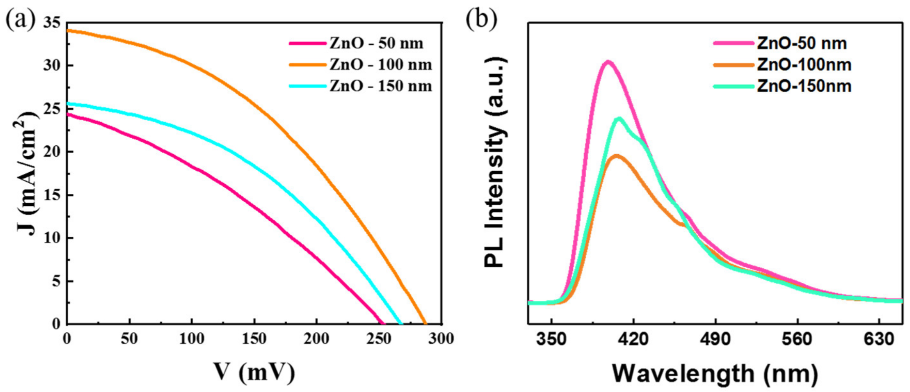

Figure S2. (a) J-V curves of the flexible CZTSSe solar cells with different thick ZnO layers. (b) PL spectra of ZnO layers with different thickness.

Table S1. GIXRD results of ZnO films annealed at different temperatures

**Temperature (℃)**	**2-Theta (°)**	**Height**	**FWHM (°)**
150	34.26	5353	0.588
	62.70	1066	0.757
200	34.32	6440	0.575
	62.80	1688	0.745
250	34.34	5618	0.556
	62.82	1475	0.710
